# Editorial: Advancements in Lung Cancer Precision Oncology Research and Treatments

**DOI:** 10.3390/biomedicines14071537

**Published:** 2026-07-09

**Authors:** Panagiotis Paliogiannis, Francesca Colonese, Giuseppe Palmieri

**Affiliations:** 1 Department of Medicine, Surgery and Pharmacy, University of Sassari, 07100 Sassari, Italy; gpalmieri@uniss.it; 2 Medical Oncology Unit, Fondazione IRCCS San Gerardo dei Tintori di Monza, 20900 Monza, Italy; francesca.colonese@irccs-sangerardo.it; 3 Unit of Cancer Genetics, Institute of Genetic and Biomedical Research (IRGB), National Research Council (CNR), 07100 Sassari, Italy

Lung cancer is one of the most common malignancies and the primary cause of cancer death worldwide [1]. It is currently estimated that less than 21% of lung cancer patients are alive five years from diagnosis; this depends on several factors, including the silent clinical course of the disease leading to late diagnosis, advanced age, respiratory impairment, cardiovascular and metabolic comorbidities, histology, and others [2]. Significant improvements in lung cancer survival have been obtained in the last decade by introducing two novel therapeutic approaches—immunotherapy and gene-targeted therapy—for patients affected by non-small cell lung cancer (NSCLC), a histological subtype that includes approximately 85% of lung cancers. These treatments are based on identifying predictive biomarkers, revolutionizing daily practice in modern pathology and medical oncology, and bridging consistent survival advantages in NSCLC patients [3]. Currently, EGFR, KRAS, BRAF, ERBB2 (HER2), ALK, ROS1, MET, RET, NRG1 and NTRK genetic alterations, as well as PD-L1 immunohistochemical expression, are used to make treatment decisions, and a great number of ongoing clinical trials investigate novel molecular targets, medications, treatment combinations and protocols against lung cancer [4,5]. To better classify such genetic heterogeneity, access to extensive molecular profiling is becoming imperative for all patients with NSCLC. As a consequence, the diagnosis and management of NSCLC is becoming more complicated with the development of newer and more specific therapies for the different molecular subtypes.

Nevertheless, durable clinical responses are currently seen only in a subset of patients, while most do not respond to treatment or relapse after an initial response. Responses to immunotherapy are shaped by both genetic and environmental factors (smokers present a higher tumor mutational load, with more chances to be responsive to immune checkpoint inhibitors) and by other immunomodulatory treatments such as radiotherapy, chemotherapy, and several targeted therapies [6,7]. Protocols of neoadjuvant chemoimmunotherapy followed by surgery in resectable stage III or chemoradiation treatment in unresectable stage III, in both cases combined with adjuvant consolidative immunotherapy, have contributed to further improving outcomes for quite a large group of NSCLC patients, though the long-term benefit on overall survival still remains unsatisfactory. Considering this evolving landscape, we have conceived this Special Issue aimed at exploring recent advances in precision medicine and immunotherapy, as well as the underlying mechanisms of treatment resistance and potential strategies to overcome them in patients with lung cancer.

Seven noteworthy articles, encompassing both translational and clinical research, are published in this collection, including six original research articles and one study report. The latter, published by Lender et al., is an innovative computational project investigating whether lung adenocarcinomas could be stratified according to their immune landscape rather than solely based on oncogenic driver alterations [8]. By applying unsupervised machine-learning algorithms to RNA sequencing-derived immune profiles, they identified three distinct immunological clusters within the lung adenocarcinoma population. Notably, one cluster was significantly enriched for EGFR-mutant tumors, suggesting that these cancers may harbor a unique immune microenvironment. Furthermore, the identified subgroups displayed significant differences in the expression of key immune checkpoint molecules, including PD-1, PD-L1, CTLA-4, and TIGIT, highlighting substantial heterogeneity in tumor–immune interactions. These findings support the concept that EGFR-mutant lung adenocarcinomas are not immunologically homogeneous and may comprise biologically distinct entities characterized by different patterns of immune evasion. Such observations are particularly relevant given the well-recognized limited efficacy of immune checkpoint inhibitors in patients with EGFR-driven disease [9,10]. By contrast, a similarly distinct immunological stratification was not observed among KRAS-mutant tumors, underscoring the complexity and diversity of KRAS-associated immune phenotypes [11]. Overall, this study provides valuable insights into the interplay between oncogenic drivers and the tumor immune microenvironment and suggests that immune-based molecular classification may further refine patient stratification and potentially guide the development of more effective immunotherapeutic strategies in lung adenocarcinoma.

Regarding the original research articles, the first study addressed several unresolved questions regarding the integration of immunotherapy into the management of advanced NSCLC [12]. Through a nationwide Delphi survey involving Italian thoracic oncologists, the authors sought to evaluate expert consensus on key clinical issues, including first-line treatment selection, the management of special patient populations, and the feasibility of immunotherapy rechallenge. The study highlighted both areas of agreement and persistent uncertainty within the oncology community. Strong consensus emerged regarding the central role of comprehensive molecular profiling in therapeutic decision-making, as well as the relevance of oncogenic alterations, smoking history, concomitant corticosteroid use, and selected scenarios for immunotherapy rechallenge. At the same time, the large number of statements failing to achieve consensus underscored the complexity of treatment decisions in contemporary NSCLC and reflected the limited availability of robust evidence for several clinically relevant situations. Importantly, the findings provide a valuable snapshot of real-world expert opinion revealing critical knowledge gaps that continue to challenge clinical practice. These unresolved issues, particularly those related to patient selection and the optimal use of immune checkpoint inhibitors across diverse clinical settings, represent important priorities for future prospective research.

The second original research article focused on a different but crucial aspect of precision oncology in NSCLC: the rapid and reliable detection of actionable EGFR alterations [13]. Given the increasing importance of molecular profiling for treatment selection and the frequent limitations imposed by small biopsy or cytology specimens [14], the authors evaluated the performance of a commercially available RT-qPCR-based assay in a large real-world cohort of patients with non-squamous NSCLC. Analyzing more than 800 consecutive clinical samples, the study showed a high concordance between the EasyPGX^®^ ready EGFR assay and next-generation sequencing (NGS), confirming the assay’s ability to accurately identify the most clinically relevant EGFR variants across a broad spectrum of specimen types and molecular characteristics. Importantly, the assay maintained excellent performance even when applied to samples with low tumor cellularity and minimal DNA input, conditions that frequently challenge routine molecular diagnostics. These findings have important practical implications. As comprehensive genomic profiling becomes increasingly integrated into clinical decision-making, the availability of rapid, accurate, and tissue-sparing diagnostic approaches is essential to ensure timely access to targeted therapies. By demonstrating reliable performance even in specimens with limited nucleic acid availability, this study highlights a potentially valuable strategy for optimizing molecular testing workflows and improving the management of patients with EGFR-driven NSCLC in real-world clinical practice.

The study by Krstić et al. represents another real-world clinically relevant contribution examining the role of consolidation durvalumab in patients with unresectable stage III NSCLC treated with sequential chemoradiotherapy [15]. While the PACIFIC trial established consolidation immunotherapy following concurrent chemoradiotherapy as the standard of care for this patient population, evidence regarding its effectiveness after sequential chemoradiotherapy remains relatively limited [16,17,18]. In this study, the authors evaluated clinical outcomes in a cohort of patients who received durvalumab after completing sequential chemoradiotherapy and demonstrated encouraging survival results, supporting the benefit of consolidation immunotherapy beyond the highly selected populations typically enrolled in randomized clinical trials. These findings contribute to the growing body of real-world evidence suggesting that the advantages of durvalumab may extend to patients who are unable to undergo concurrent chemoradiotherapy, a scenario frequently encountered in routine clinical practice. Importantly, the study also highlighted the potential impact of treatment timing, suggesting that minimizing the interval between the completion of radiotherapy and the initiation of durvalumab may further optimize patient outcomes. As stage III NSCLC continues to represent one of the most challenging and heterogeneous disease settings, these real-world data provide valuable insights into the practical implementation of immunotherapy-based strategies and support the broader applicability of consolidation durvalumab in everyday clinical care.

The study by Mazorra et al. explores a distinctly different yet equally important area of lung cancer research, focusing on cancer vaccination and the identification of immunological biomarkers associated with clinical outcomes [19]. Specifically, the authors investigated racotumomab–alum, an anti-idiotype vaccine directed against the tumor-associated ganglioside NeuGcGM3, in patients with advanced NSCLC receiving switch maintenance therapy following first-line chemotherapy. Through a comprehensive analysis of circulating immune cell populations and soluble mediators, the study identified several immunological features associated with prolonged survival. Patients deriving greater clinical benefit exhibited a more favorable immune profile, characterized by lower frequencies of regulatory T cells and specific memory T-cell subsets, alongside increased levels of natural killer T (NKT) cells and a higher CD8+ T cell-to-Treg ratio. Moreover, unfavorable outcomes were associated with increased circulating levels of pro-tumorigenic cytokines during treatment, further emphasizing the critical role of systemic immune regulation in shaping therapeutic responses. These findings provide valuable insights into the complex interplay between cancer immunotherapy and host immunity, suggesting that peripheral immune biomarkers may help identify patients more likely to benefit from vaccine-based therapeutic strategies. Although requiring validation in larger prospective studies, this work contributes to the ongoing effort to develop personalized immunotherapeutic approaches and highlights the continued potential of cancer vaccines as a component of future treatment paradigms in advanced NSCLC.

The final two original articles shift the focus from clinical research to translational investigations, providing mechanistic insights into the molecular pathways that drive lung cancer progression and resistance to targeted therapy. In this context, the study by Fang et al. explored the role of regulated in development and DNA damage response 1 (REDD1), a stress-responsive protein whose involvement in lung adenocarcinoma has remained largely undefined [20]. The authors found that REDD1 expression was significantly upregulated in tumor tissues and further induced under hypoxic conditions, a hallmark of a tumor microenvironment associated with disease progression and therapeutic resistance. Through a series of in vitro experiments, the study revealed that REDD1 promotes key malignant phenotypes, including cellular proliferation, migration, and clonogenic potential, while simultaneously suppressing apoptosis. Importantly, silencing REDD1 effectively reversed these effects, highlighting its functional contribution to tumor aggressiveness. Mechanistically, the observed biological changes appeared to be mediated, at least in part, through the ERK and JNK signaling pathways, both of which are well-established regulators of cancer cell survival and adaptation to environmental stress. Taken together, these findings provide novel evidence supporting the oncogenic role of REDD1 in lung adenocarcinoma and suggest that targeting hypoxia-driven molecular networks may represent a promising strategy for future therapeutic development.

The final contribution of this Special Issue addressed one of the most pressing challenges in the era of targeted therapies for EGFR-mutant NSCLC: the emergence of acquired resistance to osimertinib. Despite the remarkable clinical efficacy of this third-generation EGFR tyrosine kinase inhibitor, disease progression remains virtually inevitable for most patients, underscoring the need to identify novel therapeutic strategies capable of overcoming resistance mechanisms [21,22]. Using experimentally generated osimertinib-resistant cell models, the authors investigated the biological activity of AX-0085, a dual inhibitor targeting AXL and FGFR1, two signaling pathways increasingly recognized as key mediators of therapeutic escape in EGFR-driven lung cancer [23]. Through transcriptomic and functional analyses, the study demonstrated that AX-0085 effectively suppressed the activation of both receptors, modulated multiple downstream oncogenic signaling networks, and restored sensitivity to osimertinib in resistant cells. Importantly, dual AXL/FGFR1 inhibition significantly impaired several hallmarks of tumor aggressiveness, including cellular proliferation, clonogenic growth, and migratory capacity. These findings provide further evidence that bypass signaling pathways play a critical role in the development of resistance to EGFR-targeted therapies and support the rationale for combination approaches aimed at simultaneously targeting multiple resistance mechanisms. Overall, this study offers valuable preclinical insights into the complex biology of osimertinib resistance and identifies dual AXL and FGFR1 inhibition as a promising therapeutic strategy warranting further investigation. As resistance to targeted therapies continues to represent a major unmet clinical need, translational studies such as this are essential for bridging the gap between mechanistic discoveries and the development of next-generation treatment paradigms for patients with EGFR-mutant NSCLC.

Collectively, the contributions included in this collection provide a comprehensive overview of the rapidly evolving landscape of lung cancer research, spanning both clinical and translational investigations. The articles highlight the remarkable progress achieved in precision oncology and immunotherapy, while simultaneously underscoring the challenges that continue to limit long-term clinical benefit, including tumor heterogeneity, therapeutic resistance, and the identification of reliable predictive biomarkers. Importantly, the studies presented herein emphasize the value of integrating molecular characterization, immune profiling, and mechanistic research to further refine patient selection and develop more effective therapeutic strategies. Taken together, we believe that these contributions advance our understanding of the molecular and immunological mechanisms underlying lung cancer and may help inform future strategies for research.

## Data Availability

No new data were created in this study.
